# Identification of SARS-CoV-2 E Channel Blockers from a Repurposed Drug Library

**DOI:** 10.3390/ph14070604

**Published:** 2021-06-23

**Authors:** Prabhat Pratap Singh Tomar, Miriam Krugliak, Isaiah T. Arkin

**Affiliations:** Department of Biological Chemistry, The Alexander Silberman Institute of Life Sciences, The Hebrew University of Jerusalem, Edmond J. Safra Campus Givat-Ram, Jerusalem 91904, Israel; ppstdbt@gmail.com (P.P.S.T.); miriamkru@savion.huji.ac.il (M.K.)

**Keywords:** COVID-19, viral channels, bacterial assays, channel blockers, antiviral drugs

## Abstract

SARS-CoV-2, the etiological agent of the COVID-19 pandemic, is a member of the Coronaviridae family. It is an enveloped virus with ion channels in its membrane, the most characterized of which is the E protein. Therefore, in an attempt to identify blockers of the E channel, we screened a library of 2839 approved-for-human-use drugs. Our approach yielded eight compounds that exhibited appreciable activity in three bacteria-based channel assays. Considering the fact that the E channel is the most conserved of all SARS-CoV-2 proteins, any inhibitor of its activity may provide an option to curb the viral spread. In addition, inhibitors can also enhance our ability to understand the exact role played by the E protein during the infectivity cycle. Finally, detailed electrophysiological analyses, alongside in vitro and in vivo studies will be needed to establish the exact potential of each of the blockers identified in our study.

## 1. Introduction

At the end of 2019, a new respiratory disease spread across much of the globe. Approximately 100 million people were found to be infected by the virus in a year, with a mortality rate exceeding 2% [1]. The pandemic’s etiological agent was quickly identified as a new coronavirus [2,3] and was found to be very similar to the virus that caused the SARS epidemic in 2002/3 [4,5]. Accordingly, the virus was named SARS-CoV-2 [6].

As a member of the Coronaviridae family, SARS-CoV-2 is an enveloped virus and contains several proteins in its membrane. One of the membrane constituents is the E protein, which has been implicated in viral assembly, release, and pathogenesis, based on studies on other coronaviruses [7]. Notably, E proteins were shown to be essential for viral infectivity [8], and attenuated viruses that lack them were suggested to serve as vaccine candidates [9,10,11,12].

The structure of the transmembrane domain of SARS-CoV-2 E protein was recently determined by solid-state NMR spectroscopy [13]. The pentameric channel bundle deviates from ideal α-helicity and surrounds a dehydrated pore.

Functionally, E proteins from several coronaviruses, including the very similar SARS-CoV-1, were shown to possess cation-selective channel activity [14,15,16]. Consequently, we have recently confirmed that the E protein from SARS-CoV-2 is also a channel using bacteria-based assays [17].

As a protein family, ion channels serve as excellent and frequent targets for pharmaceutical point interventions. For example, chemicals that manipulate ion channels are used to treat many diseases such as cystic fibrosis, epilepsy, arrhythmia, neurodegenerative diseases, hypertension, angina, and more [18].

Ion channels in viruses have also been suggested to serve as attractive drug targets [19,20]. However, currently only one class of channel blockers are approved as antiviral agents: the antiflu aminoadamantane drugs [21] that target influenza’s M2 protein [22] by blocking its channel activity [23]. Regrettably, widespread resistance by the virus (due to poor genomic replication fidelity) has rendered aminoadamantanes ineffective [24].

Considering the above, we have decided to search for blockers against the SARS-CoV-2 E protein channel. Such blockers may present a potential approach to curb infectivity, in particular considering the fact that the E protein is the most conserved of all viral proteins [2,3]. For example, while the spike proteins of SARS-CoV-2 and SARS-CoV-1 are only 76.2% identical, their respective E proteins are 93.5% identical [2]. In addition, channel blockers can also serve as useful research tools to elucidate the role of the E protein in the viral infectivity cycle.

In preliminary studies employing a small library of 372 channel blockers, we identified two low-affinity inhibitors of the protein [17], motivating further screening efforts. Therefore, in the current study, we focussed our search on a significantly larger library of 2839 approved-for-use compounds. Drug repurposing as such entails two benefits: minimizing the chemical space to explore and potentially expediting any future regulatory steps.

## 2. Results

Our screening strategy employed the analysis of bacteria that heterologously express the E protein and consequently exhibit a specific phenotype. Subsequently, the effect of different chemicals on the channel may be examined by monitoring their impact on the bacteria by reversing said phenotype.

To ensure proper membrane reconstitution, we used the MBP Fusion and Purification System (New England BioLabs, Ipswich, MA, USA), in which the E protein was fused to the carboxy terminus of the maltose-binding protein. Utilizing this construct, we have recently shown that SARS-CoV-2 E protein is expressed in a functional form in bacteria [17]. Finally, this system has been used to express and study numerous viral ion channels successfully, such as M2 from the influenza virus, Vpu from HIV, 6k from the Eastern equine encephalitis virus, MgM from the West Nile virus, 2k and P1 from the Dengue virus, gp170 and gp151 from the Variola virus, and 3a from SARS-CoV-2 [25,26,27,28,29,30].

### 2.1. Negative Assay

The first assay that we used is one in which the viral protein is expressed at elevated levels in the bacteria. Consequently, the bacteria experience severe growth retardation due to excessive membrane permeabilization caused by the viral channel. In other words, the viral channel impacts the bacteria *negatively*. As a result, blockers of the viral channel may be identified due to their ability to revive bacterial growth.

Using the aforementioned approach, we screened 2839 chemicals from the drug repurposing library of MedChem Express (Monmouth Junction, NJ, USA) (note that the number of compounds in the library increases with time). Specifically, bacterial cultures were grown overnight in 96-well plates, and the impact of each chemical in the library at a concentration of 100 µM was tested individually. Finally, any hit was then analyzed at several different concentrations to obtain a dose–response curve.

**Figure 1:**
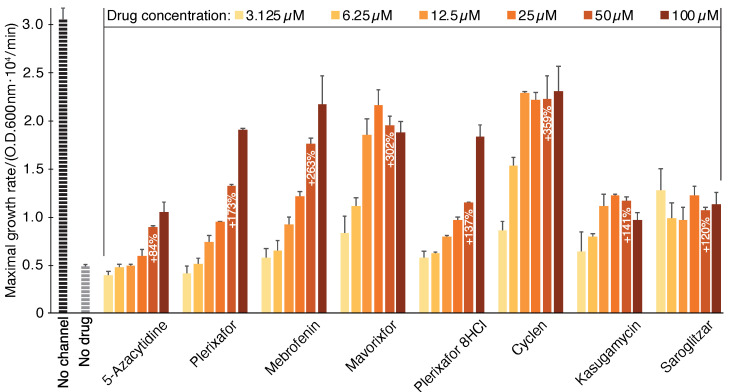
Compound screening results using the negative assay. SARS-CoV-2 E protein is expressed at an elevated level (induction with 100 µM [β-d-1-thiogalactopyranoside]) and is therefore deleterious to bacteria. In this instance, inhibitory drugs enhance bacterial growth. The results may be compared to those obtained without any drug (gray) or when the channel is uninduced (black). The color scale indicates the different concentrations of the chemicals.

**Figure 2:**
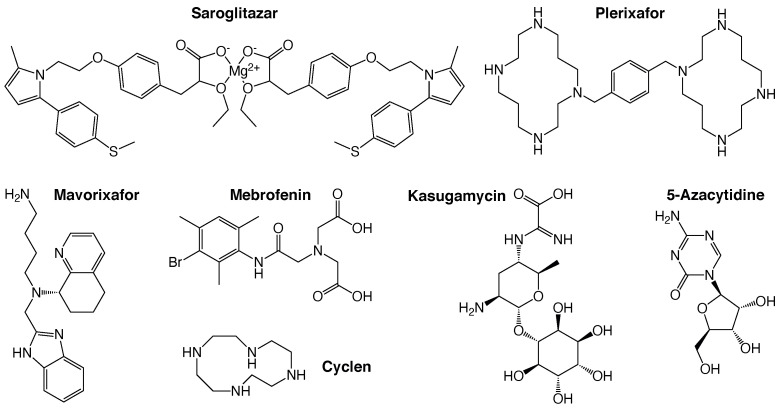
Chemical structures of the hits identified in the study. Note that the only the uncomplexed form of Plerixafor is shown.

The results shown in Figure 1 indicate that the following eight drugs are able to revive bacterial growth to varying extents: 5-Azacytidine (+84%), Plerixafor (+173%), Mebrofenin (+263%), Mavorixafor (trihydrochloride) (+302%), Plerixafor (octahydrochloride) (+137%), Cyclen (+359%), Kasugamycin (hydrochloride hydrate) (+141%), and Saroglitazar Magnesium (+120%). The values in parenthesis are the growth enhancement of each chemical at 50 µM relative to untreated bacteria. Finally, see panels a and b of Supplementary Figures S1–S8 for raw growth curves of each of the compounds, alongside detailed chemical structures shown in Figure 2.

We recognize the potential of spurious factors to impact bacterial growth, leading to false identification of hits. Therefore, each chemical that scored positively in the negative assay was tested in a reciprocal assay, and in doing so, fallacious results are minimized significantly.

### 2.2. Positive Assay

The second bacterial assay that we used is one in which the viral protein is expressed at low levels in K^+^-uptake deficient bacteria. These bacteria are incapable of growth unless the media is supplemented by K^+^ [31]. However, when a channel capable of K^+^ transport is heterologously expressed, the bacteria can thrive even in low K^+^ media [27,28]. Hence, in this instance, the viral channel *positively* impacts the bacteria, and channel blockers result in growth retardation. This scenario is entirely reciprocal to the negative assay described above in Section 2.1, and therefore serves to verify its results.

**Figure 3:**
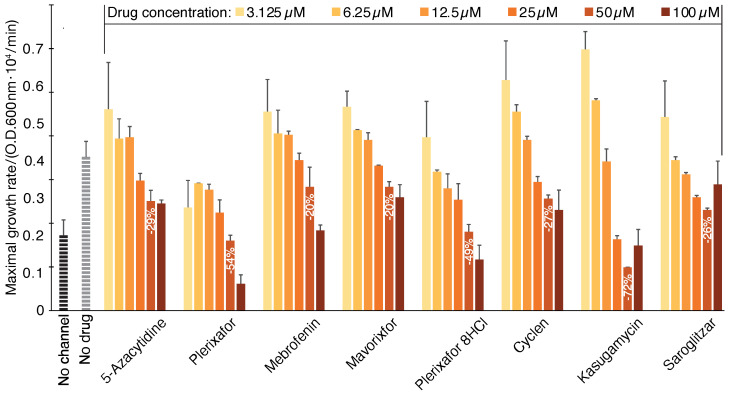
Compound screening results using the positive assay. SARS-CoV-2 E protein is expressed at a low level (20 µM [β-d-1-thiogalactopyranoside]) in K^+^-uptake deficient bacteria. In this instance, inhibitory drugs reduce bacterial growth. The results may be compared to those obtained without any drug (gray) or when the channel is uninduced (black). The color scale indicates the different concentrations of the chemicals.

Each of the hits identified in the negative assay was subjected to a dose–response analysis using the positive assay, as depicted in Figure 3. The results present a mirror image of the negative assay (Figure 1), whereby in this instance the compounds decreased growth as follows: 5-Azacytidine (−29%), Plerixafor (−54%), Mebrofenin (−20%), Mavorixafor trihydrochloride (−20%), Plerixafor octahydrochloride salt (−49%), Cyclen (−27%), Kasugamycin hydrochloride hydrate (−72%), and Saroglitazar Magnesium (−26%). The values in parenthesis are the growth reduction of each chemical at 50 µM relative to untreated bacteria. Detailed growth curves of each compound can be found in panels c and d of Supplementary Figures S1–S8.

### 2.3. Fluorescence-Based Test

The final test to examine the activity of channel blockers is based on detecting protein-mediated H^+^ flux. Bacteria that express a chromosomally encoded pH-sensitive green fluorescent protein [32] exhibit fluorescence changes when their internal pH is altered. In particular, adding an acidic solution to the media will result in a readily detectable fluorescence change due to cytoplasmic acidification if the bacteria express a channel capable of H^+^ transport [33]. Therefore, in this assay, blockers may be identified by their ability to diminish the channel-driven fluorescence change.

**Figure 4:**
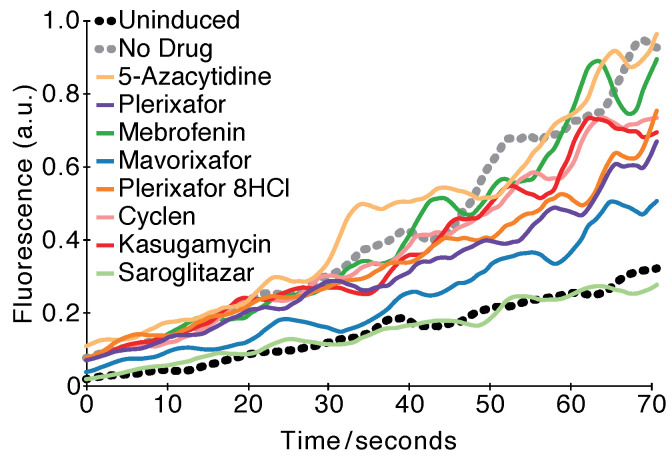
Fluorescence-based conductivity assay. The fluorescence of bacteria that harbor a pH-sensitive GFP [32] and express the SARS-CoV-2 E protein was examined as a function of different chemicals at a concentration of 100 µM. The experiment was performed as previously described [33], whereby at time 0, a concentrated solution of citric acid was injected into the media. The results may be compared to those obtained without any drug (black) or when the channel expression was not induced (gray).

As seen in Figure 4, most of the compounds are able to reduce the viroporin-induced fluorescence change with the exception of 5-Azacytidine and Mebrofenin. Saroglitazar was able to suppress the change entirely, while the impact of Cyclen was minor. Results at several drug concentrations including error bars can be found in panel e of Supplementary Figures S1–S8.

## 3. Discussion

Channel blockers are powerful agents found in the natural world. Tetrodotoxin and saxitoxin, for example, are present in marine organisms and are potent blockers of sodium channels, thereby underpinning their hosts’ toxicity [34]. Pharmaceutical intervention has also been very successful in utilizing and synthesizing channel blockers for numerous indications. Prime examples of such compounds are the dihydropyridine [35], phenylalkylamine [36], and benzothiazepine [37] calcium channel blockers.

Similarly, blockers of viral ion channels hold the promise of providing an approach to curb infectivity, alongside serving as useful research tools [19]. Since E proteins are essential components of coronaviruses [8], as well as being the most conserved protein in SARS-CoV-2 [2], any inhibitor against them would be particularly useful.

In order to identify inhibitors against the E protein, we decided to search amongst agents that are already approved for human use. Repurposing as such has proven to be a reliable route towards drug discovery, in general, and for identifying antiviral drugs in particular. For example, the repurposing of azidothymidine (AZT) to combat AIDS [38,39] was reported more than twenty years after its first description in 1964 [40].

Repurposing also represents one of the fastest approaches to curbing infectivity [41]. As an example, the only antiviral drug that is currently approved against COVID-19 is a product of repurposing—remdesivir. While its efficacy may still be a matter of contention [42,43,44], it is nonetheless an example of the speed at which drug repurposing can react to a health crisis.

Considering the above, it is not surprising that there have been numerous repurposing studies against SARS-CoV-2. While the vast majority have employed in silico screenings, others have taken an experimental route. For example, Riva et al. screened 12,000 clinical-stage or FDA-approved drugs for their ability to inhibit viral replication [45]. The encouraging results of this monumental study were 21 molecules that exhibited a dose–response activity profile.

Herein, a different and potentially complementary approach was taken, focussing on a single target of the virus—the E protein. Our rationale stemmed from the fact that channels are attractive drug targets, and searching for inhibitors against them is both rapid and economically viable in an academic setting. Furthermore, genetic selections in bacteria may cast a wider net when targeting an individual protein due to the host’s higher toxicity tolerance. Such studies also open the door to mutational analyses that may provide insight into protein function and drug resistance mechanisms [25].

Three independent bacteria-based assays were used to search the repurposed drug library. The first two tests are reciprocal, whereby in the negative assay, the channel is detrimental to bacterial growth, while in the positive assay, it is beneficial. Consequently, blockers will yield the opposite outcomes in both assays: in the negative assay, they will enhance growth, while in the positive assay, they will retard it. The use of two assays minimizes any erroneous hits: the negative assay is susceptible to any pleiotropic growth enhancers’ activity, leading to false positives. Similarly, the positive assay would score a hit for any toxic compound. Yet, it is difficult to imagine how a drug can enhance the growth of bacteria in the negative assay while at the same time retard them in the positive assay if its effect was not specific. Finally, it is important to note that this combination of assays has been successfully validated using other viral channels such as Influenza M2 [25,26,30] and HIV Vpu [27]. In both of these instances, known inhibitors enhanced bacterial viability in the negative assay while diminishing growth in the positive assay.

The outcome of both tests comprised the list of compounds to be tested for antiviral activity. However, a third, fluorescence-based assay was employed to provide potential validation for the hits. In this test, a pH-sensitive GFP can report the change in the cytoplasm’s acidity [32]. Subsequently, the activity of a channel that is capable of H^+^ transport can be detected by measuring the fluorescence change due to acidification of the external media. Consequently, channel blockers would diminish the fluorescence change, leading to their identification.

Gratifyingly, most of the hits identified by the positive and negative assays were able to lower the fluorescence change, with the exception of 5-Azacytidine and Mebrofenin (Figure 4). Since non-specific factors influencing pH may obfuscate detection, we decided not to eliminate the latter two chemicals from our hits list to be as inclusive as possible.

When analyzing the outcomes of the screening tests, it is essential to realize that the assays are bacteria-based and, as such, should not be compared quantitatively to one another. For example, the negative assay measures the detrimental impact of channel overexpression on bacteria due to membrane permeabilization. On the other hand, the positive assay measures K^+^ conductivity, which is essential for the K^+^-uptake deficient bacteria to survive. Hence, for screening purposes, any chemical that passed the positive and negative assay, regardless of ranking, was designated as a hit.

None of the compounds that the current screen yielded were identified by the large repurposing study of Riva and coworkers [45]. One obvious factor that may explain the different outcomes between the two studies is stringency. Riva and coworkers screened every chemical at 5 µM, whereas the current research on bacteria employed 100 µM. Screening at this higher concentration stemmed from our desire to cast a wide net, which is feasible in the more tolerant bacterial system. While molecules can emerge from the bacterial screen with lower affinities, they may still be beneficial, serving as a starting point for detailed chemical exploration. Moreover, low-affinity drugs that block the E-channel may interact synergistically with inhibitors of other targets in the virus.

A comparison of the structures of the different drugs does not yield any obvious recurring element, with the possible exception of the presence of cyclic groups. While most compounds are basic (Cyclen and Plerixafor in particular), some are not. Similarly, the sizes of the chemicals vary considerably, from Cyclen (172 g/mol) to Plerixafor (794 g/mol). Electrophysiological studies may be able to uncover which recurring elements of each blocker are required for activity, facilitating the path for medicinal chemistry improvements. In this respect, drug repurposing can be viewed as a starting point for exploring a much larger chemical space.

## 4. Materials and Methods

### 4.1. Channel Assays

All three bacteria-based assays were conducted as described previously [17,28]. In brief, bacterial cultures were diluted and grown overnight until their O.D._600 nm_ reached 0.2. An amount of 50 µL of culture was subsequently transferred into 96-well flat-bottomed plates containing 50 µL of the different treatments. Protein induction was achieved by adding β-d-1-thiogalactopyranoside at 100 µM or 20 µM for the negative and positive assays, respectively. D-glucose was added to a concentration of 1%. The plates were incubated for 16 h at 37 ${}^{\circ }$C in a multi-plate incubator (Tecan Group, Männedorf, Switzerland) at a constant high shaking rate. O.D._600 nm_ readings were recorded every 15 min on an Infinite 200 plate reader (Tecan Group). For every measurement, duplicates or triplicates were conducted.

The positive assay was conducted in a similar manner, except that the K^+^-uptake deficient bacteria were grown overnight and diluted in LB media, in which Na${}^{+}$ was replaced by K^+^. Thereafter, the growth medium was replaced with LB, which was supplemented with 5 mM KCl.

The fluorescence-based assay was conducted with bacteria that harbor a chromosomal copy of a pH-sensitive GFP [33,46]. Overnight cultures were diluted to 1:500 in LB media and grown up to an O.D._600 nm_ of 0.6–0.8. E protein expression was then induced by adding 50 µM β-d-1-thiogalactopyranoside to the growth media. After one hour of induction, the O.D._600 nm_ of all cells was measured, and after pelleting at 3500 g for 10 min, the bacteria were resuspended in McIlvaine buffer (200 mM Na${}_{2}$HPO${}_{4}$, 0.9%NaCl adjusted to pH 7.6 with 0.1 M citric acid, 0.9%Nacl) to an optical density of 0.25 at 600 nm. Then, 200 µL of cell suspension was subsequently transferred with 30 µL of McIlvaine buffer to 96-well plate. The plate includes a row with only assay buffer and cultures without induction. The fluorescence measurement were carried out on an Infinite F200 pro microplate reader (Tecan Group, Männedorf, Switzerland).

At time zero, 70 µL of 300 mM Citric acid with 0.9% NaCl was added to the bacteria. The fluorescence emission of each well after addition of acid was measured by an alternate read out of the two wavelengths for 30 s. The ratio for the two differently excited emissions, $F=F_{390\;\mathrm{nm}}/F_{466\;\mathrm{nm}}$, was calculated and translated into proton concentration according to [33,46].

### 4.2. Chemical Screening

A library of 2839 repurposed drugs was purchased from MedChem Express (HY-L035, Monmouth Junction, NJ, USA). Note that the number of chemicals in the library changes with time. Each chemical was added at 100 µM concentration to the growth media with a total concentration of Dimethyl sulfoxide not exceeding 1%. All manipulations and growths were conducted on a Tecan EVO 75 robotic station (Männedorf, Switzerland).

At first, we screened all compounds in the negative assay using 96-well plates. Each plate had a positive and negative control. The positive control were bacteria in which channel expression was not induced, i.e., without β-d-1-thiogalactopyranoside. The negative control was bacteria to which DMSO was added without any chemicals.

Bacteria that exhibited growth enhancement above a certain empirical threshold were tested again in duplicate. Every compound that passed this test was then used in the positive assay in duplicate. Compounds that passed the positive and negative screens were then subjected to a dose–response analysis, as well as a fluorescence-based study.

## 5. Conclusions

Multiple, independent bacteria-based assays were able to retrieve eight compounds from a library of 2839 approved-for-human-use drugs that inhibit SARS-CoV-2 E protein. Since E protein is an essential component of the pathogen, as well as the most conserved of all SARS-CoV-2 proteins, any of its inhibitors represents a potential avenue to curb infectivity. As such, the stage is now set for in vitro and in vivo studies (in appropriate bio-safety facilities) to examine the effects of the compounds on the virus. Similarly, electrophysiological studies will be required to determine the molecular details that characterize each blocker’s activity.

## Data Availability

The data presented in this study are available on request from the corresponding author.

## Supplementary Materials

The following are available online at https://www.mdpi.com/article/10.3390/ph14070604/s1: Figures S1–S8, depicting individual growth curves of each of the compounds, chemicals structures, and fluorescence-based conductivity assay results at several different concentrations including error bars.
