# Supplementary material for: Identification of SARS-CoV-2 E Channel Blockers from a Repurposed Drug Library
Source: Pharmaceuticals (Basel). 2021 Jun 23;14(7):604. doi: 10.3390/ph14070604 (PMC8308726; doi:10.3390/ph14070604)
Supplement: Supplementary file 1 [file pharmaceuticals-14-00604-s001.zip › pharmaceuticals-1254029-supplementary.pdf]

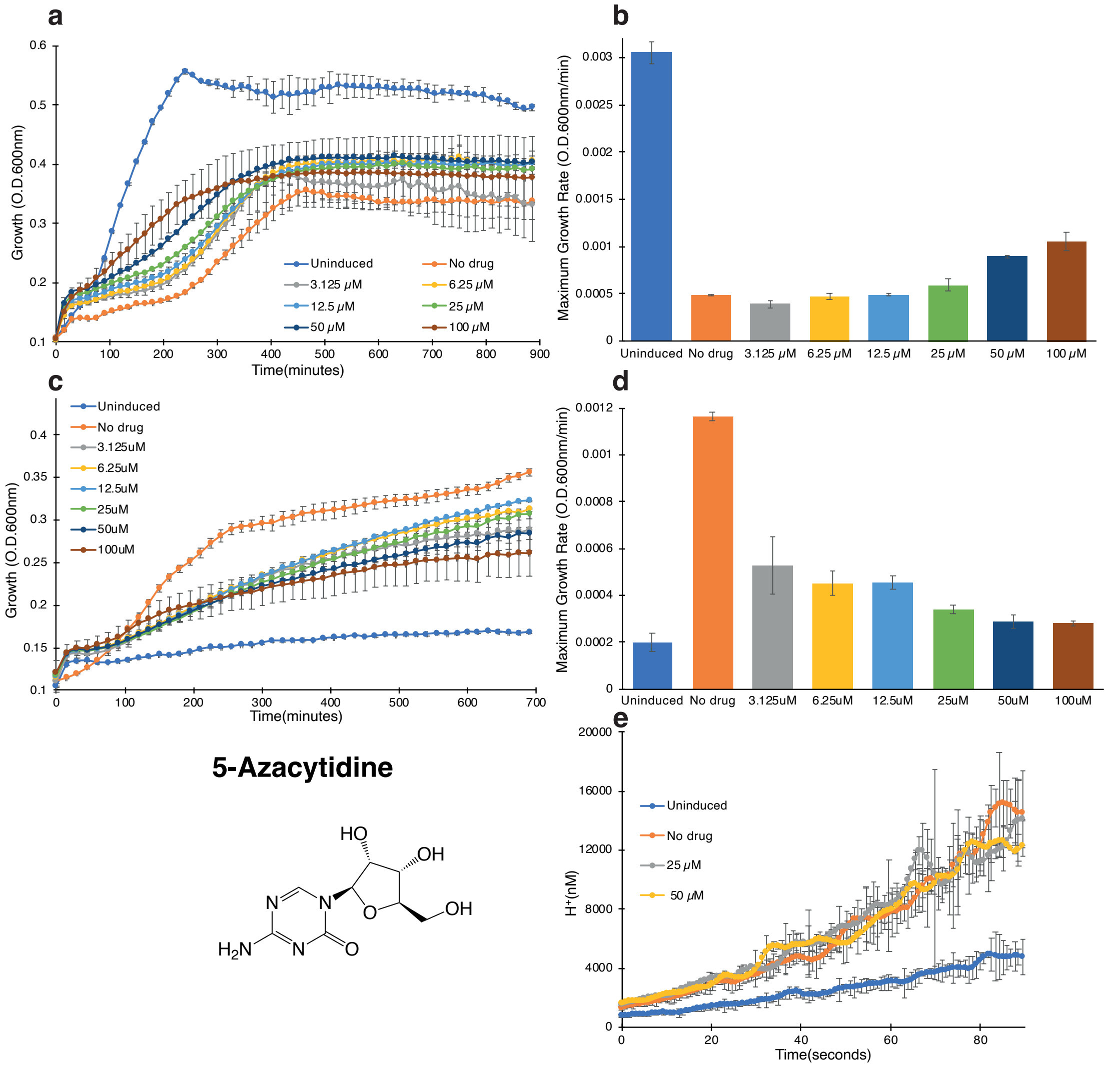

Figure S1. Raw screening data for 5-Azacytidine. a. Negative assay in which SARS-CoV-2 E protein is expressed at an elevated level (induced with 100 µM [β-D-1-thio-galactopyranoside]) and is therefore deleterious to bacteria. The different concentrations of the drug are indicated. b. Maximal growth rates obtained in the negative assay. c. Positive assay in which SARS-CoV-2 E protein is expressed at a low level (induced with 20 µM [β-D-1-thiogalactopyranoside]) in K^+-uptake deficient bacteria [31]. In this instance, inhibitory drugs reduce bacterial growth. d. Maximal growth rates obtained in the positive assay. e. Fluorescence-based conductivity assay. The fluorescence of bacteria that harbor a pH-sensitive GFP [32] and express the SARS-CoV-2 E protein was examined as a function of different chemical concnetration as noted. The experiment was performed as previously described [33], whereby at time 0, a concentrated solution of citric acid was injected into the media. In all panels LB indicates bacteria that do not express the channel as a positive control, while 100 µM IPTG indicates no drug added as a negative control.

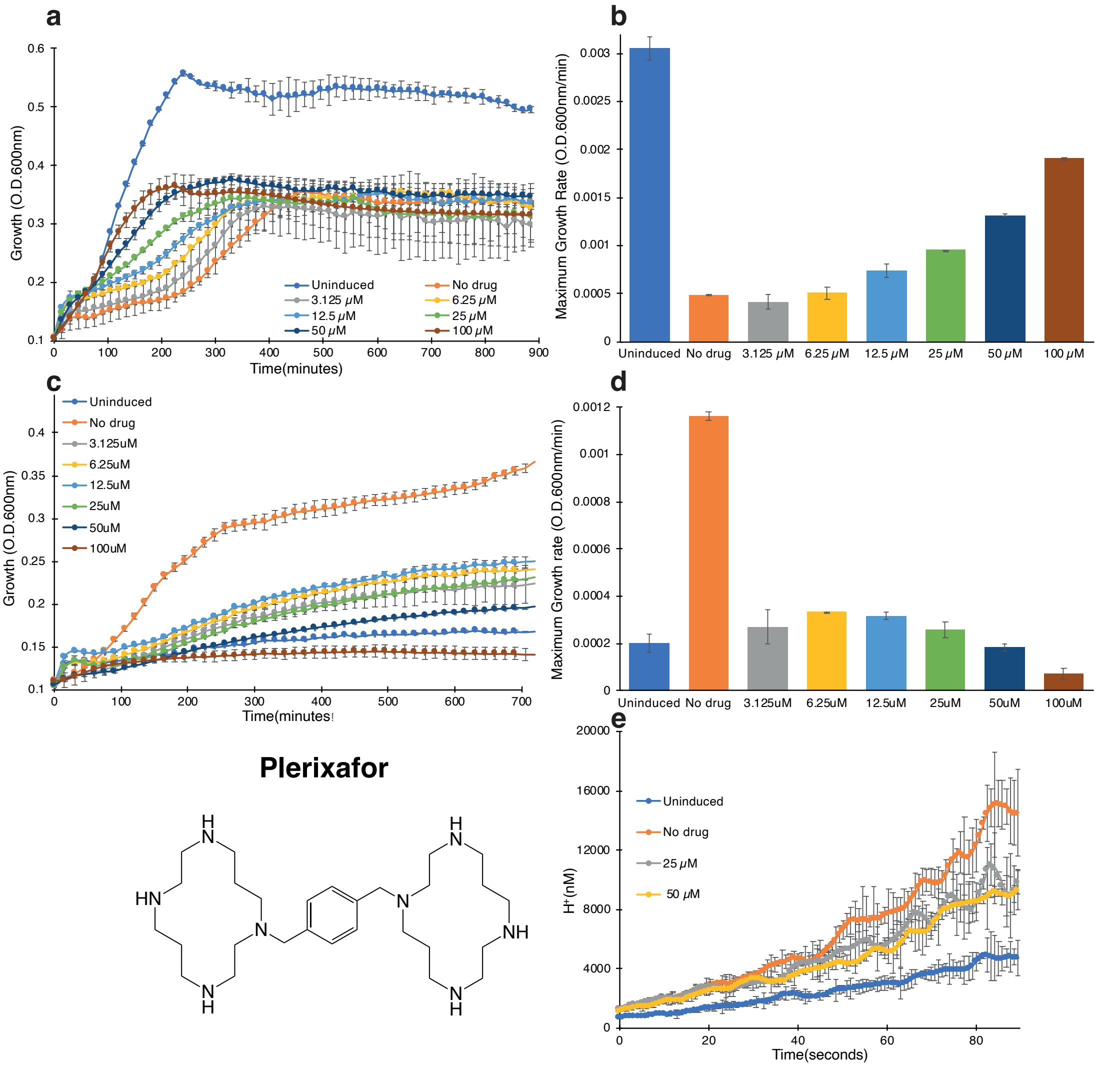

Figure S2. Raw screening data for Plerixafor. a. Negative assay in which SARS-CoV-2 E protein is expressed at an elevated level (induced with 100 μM [β-D-1-thiogalactopyranoside]) and is therefore deleterious to bacteria. The different concentrations of the drug are indicated. b. Maximal growth rates obtained in the negative assay. c. Positive assay in which SARS-CoV-2 E protein is expressed at a low level (induced with 20 μM [β-D-1-thiogalactopyranoside]) in K^+-uptake deficient bacteria [31]. In this instance, inhibitory drugs reduce bacterial growth. d. Maximal growth rates obtained in the positive assay. e. Fluorescence-based conductivity assay. The fluorescence of bacteria that harbor a pH-sensitive GFP [32] and express the SARS-CoV-2 E protein was examined as a function of different chemical concnetration as noted. The experiment was performed as previously described [33], whereby at time 0, a concentrated solution of citric acid was injected into the media. In all panels LB indicates bacteria that do not express the channel as a positive control, while 100 μM IPTG indicates no drug added as a negative control.

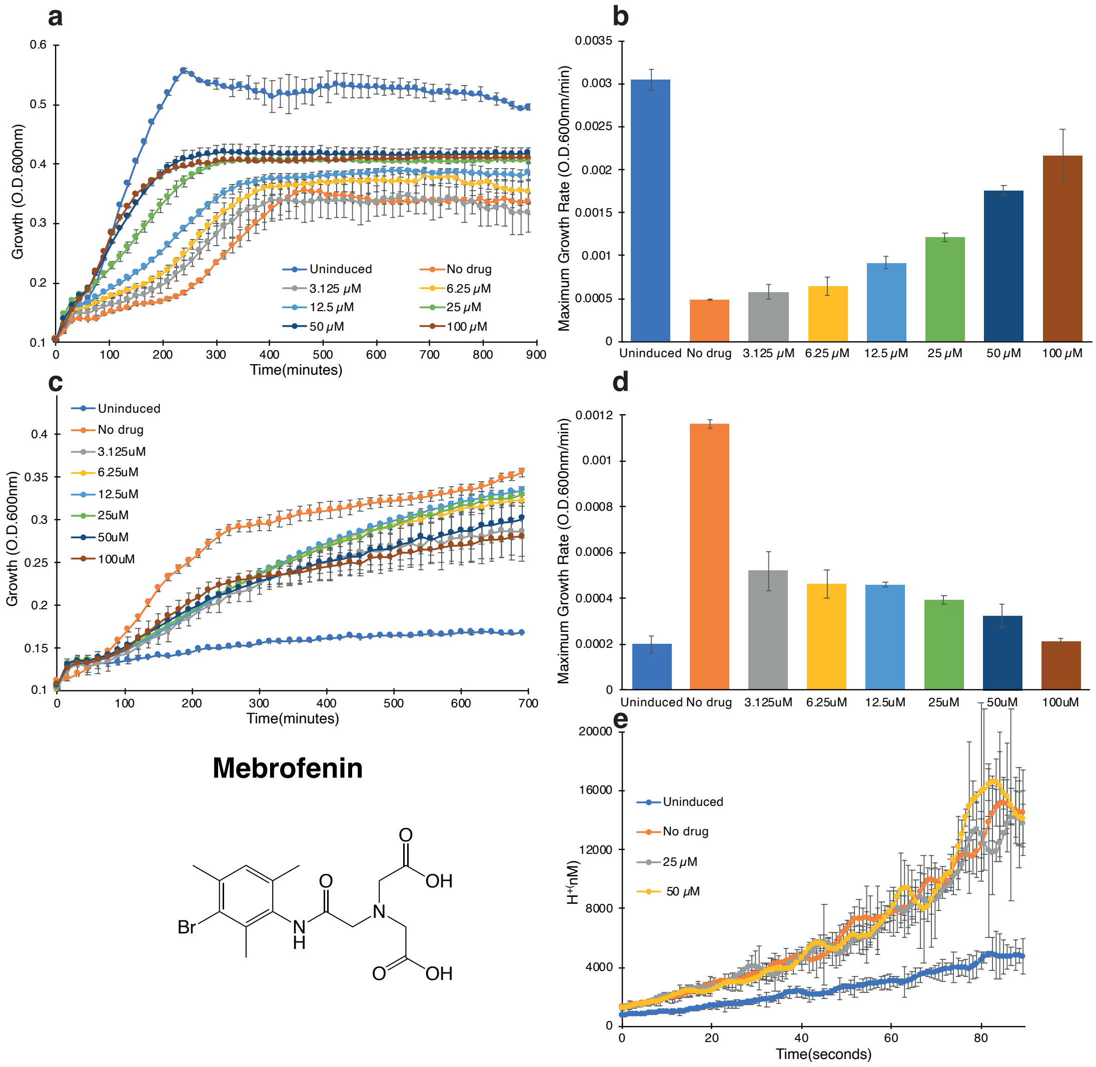

Figure S3. Raw screening data for Mebrofenin. a. Negative assay in which SARS-CoV-2 E protein is expressed at an elevated level (induced with 100 μM [β-D-1-thiogalacto-pyranoside]) and is therefore deleterious to bacteria. The different concentrations of the drug are indicated. b. Maximal growth rates obtained in the negative assay. c. Positive assay in which SARS-CoV-2 E protein is expressed at a low level (induced with 20 μM [β-D-1-thiogalactopyranoside]) in K^+-uptake deficient bacteria [31]. In this instance, inhibitory drugs reduce bacterial growth. d. Maximal growth rates obtained in the positive assay. e. Fluorescence-based conductivity assay. The fluorescence of bacteria that harbor a pH-sensitive GFP [32] and express the SARS-CoV-2 E protein was examined as a function of different chemical concnetration as noted. The experiment was performed as previously described [33], whereby at time 0, a concentrated solution of citric acid was injected into the media. In all panels LB indicates bacteria that do not express the channel as a positive control, while 100 μM IPTG indicates no drug added as a negative control.

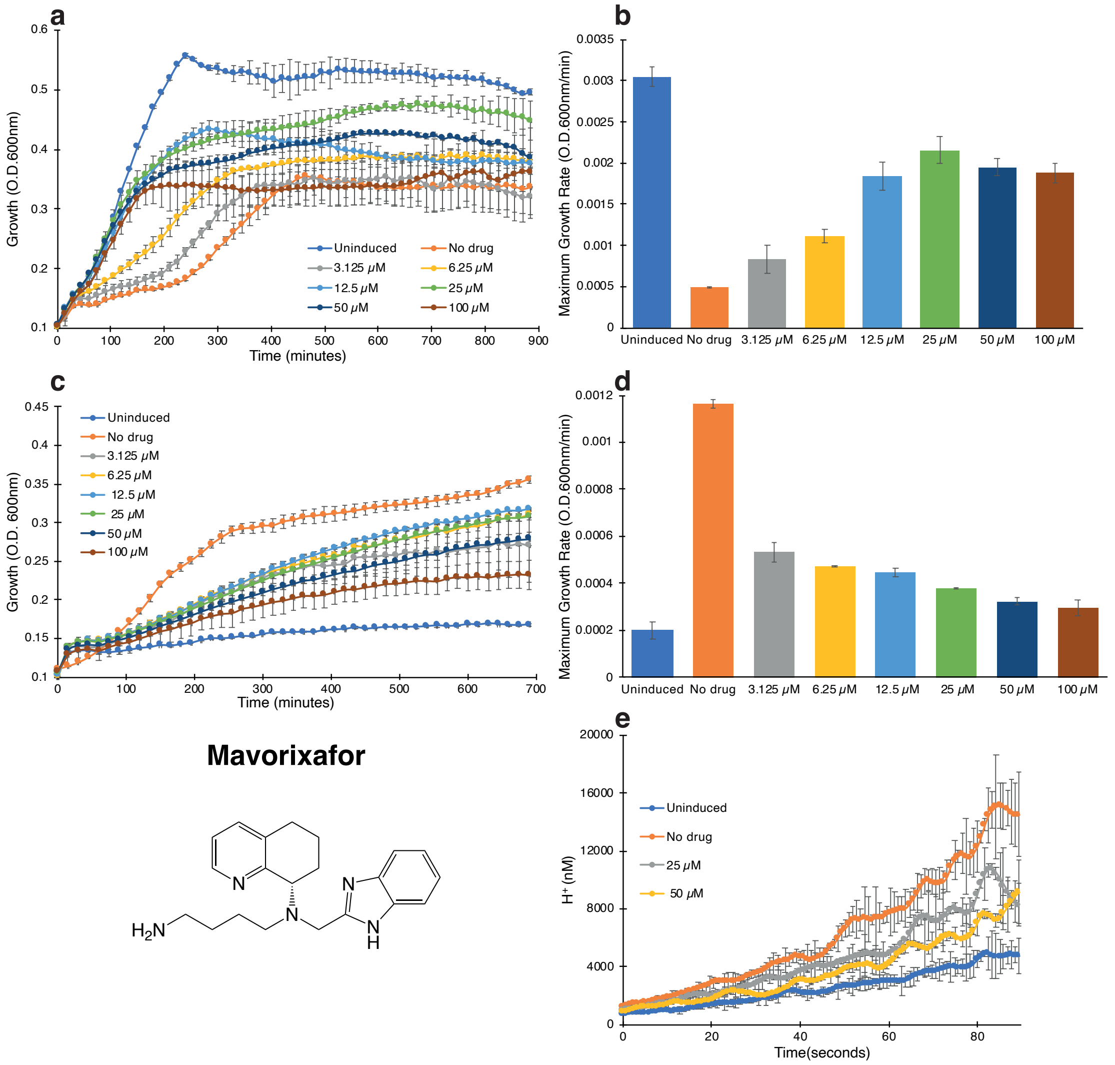

Figure S4. Raw screening data for Mavorixafor. a. Negative assay in which SARS-CoV-2 E protein is expressed at an elevated level (induced with 100 μM [β-D-1-thiogalacto-pyranoside]) and is therefore deleterious to bacteria. The different concentrations of the drug are indicated. b. Maximal growth rates obtained in the negative assay. c. Positive assay in which SARS-CoV-2 E protein is expressed at a low level (induced with 20 μM [β-D-1-thiogalactopyranoside]) in K^+-uptake deficient bacteria [31]. In this instance, inhibitory drugs reduce bacterial growth. d. Maximal growth rates obtained in the positive assay. e. Fluorescence-based conductivity assay. The fluorescence of bacteria that harbor a pH-sensitive GFP [32] and express the SARS-CoV-2 E protein was examined as a function of different chemical concnetration as noted. The experiment was performed as previously described [33], whereby at time 0, a concentrated solution of citric acid was injected into the media. In all panels LB indicates bacteria that do not express the channel as a positive control, while 100 μM IPTG indicates no drug added as a negative control.

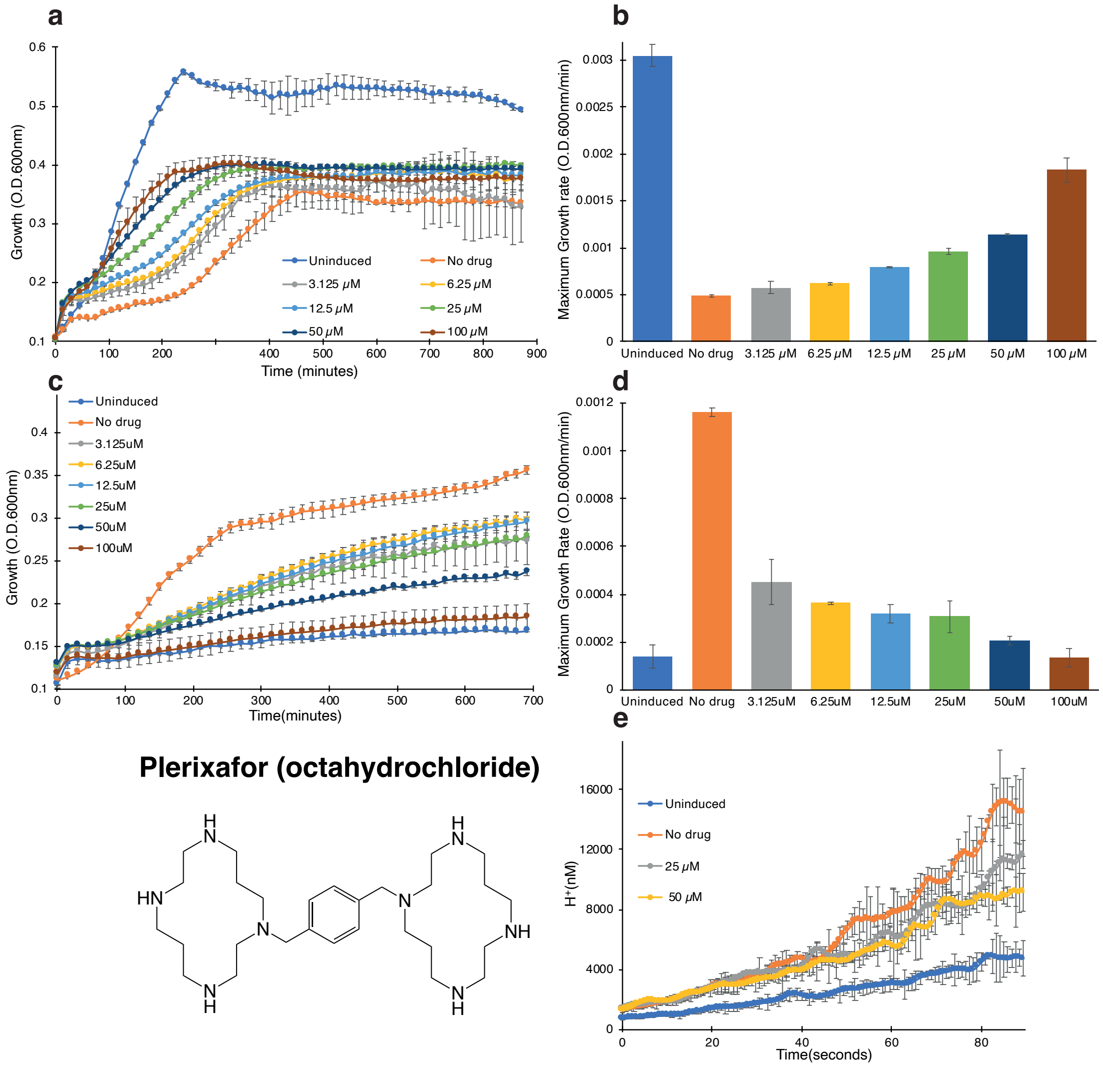

Figure S5. Raw screening data for Plerixafor (octahydrochloride). a. Negative assay in which SARS-CoV-2 E protein is expressed at an elevated level (induced with 100 μM [β-D-1-thiogalactopyranoside]) and is therefore deleterious to bacteria. The different concentrations of the drug are indicated. b. Maximal growth rates obtained in the negative assay. c. Positive assay in which SARS-CoV-2 E protein is expressed at a low level (induced with 20 μM [β-D-1-thiogalactopyranoside]) in K^+-uptake deficient bacteria [31]. In this instance, inhibitory drugs reduce bacterial growth. d. Maximal growth rates obtained in the positive assay. e. Fluorescence-based conductivity assay. The fluorescence of bacteria that harbor a pH-sensitive GFP [32] and express the SARS-CoV-2 E protein was examined as a function of different chemical concnetration as noted. The experiment was performed as previously described [33], whereby at time 0, a concentrated solution of citric acid was injected into the media. In all panels LB indicates bacteria that do not express the channel as a positive control, while 100 μM IPTG indicates no drug added as a negative control.

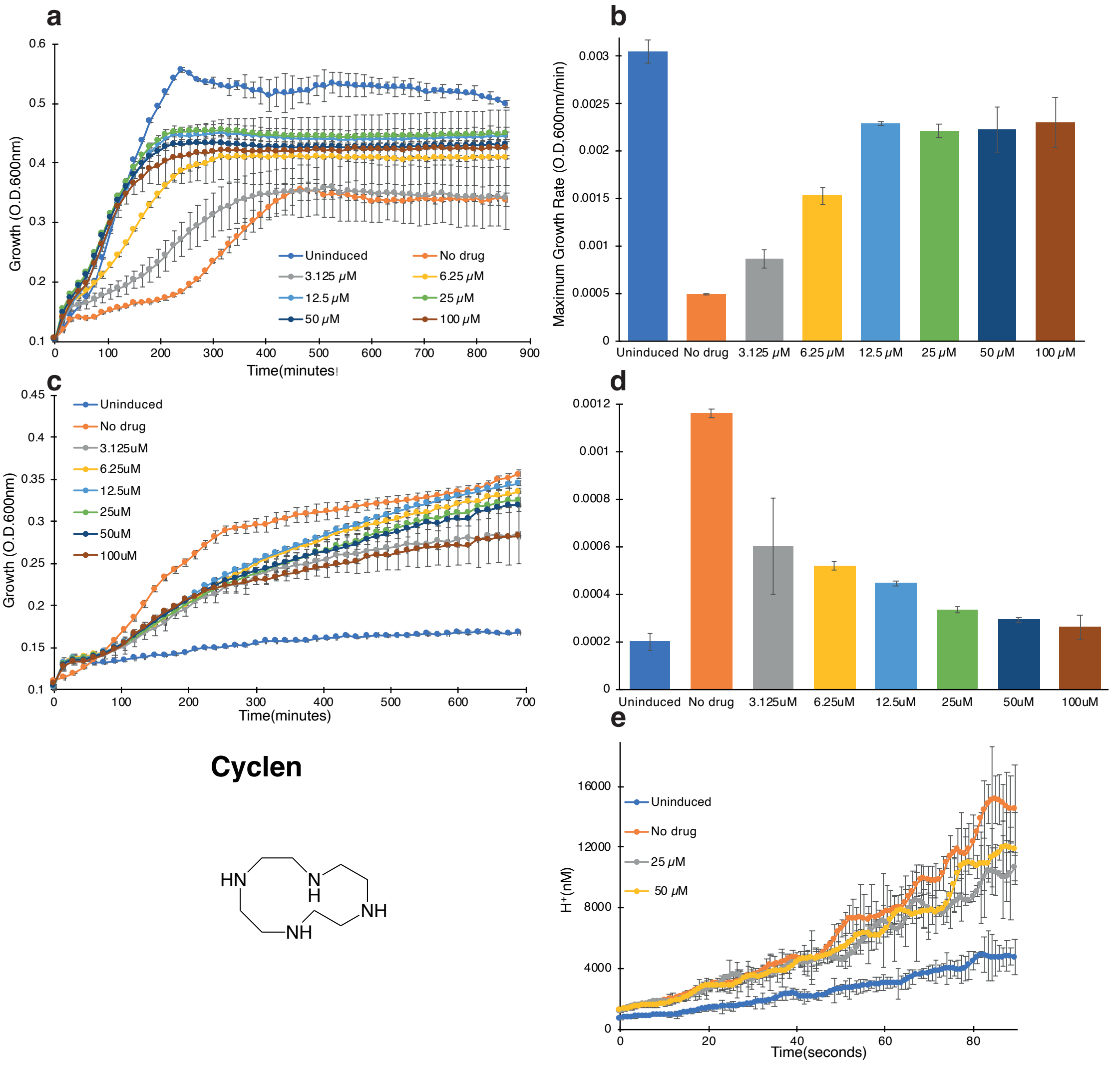

Figure S6. Raw screening data for Cyclen. a. Negative assay in which SARS-CoV-2 E protein is expressed at an elevated level (induced with 100 μM [β-D-1-thiogalacto-pyranoside]) and is therefore deleterious to bacteria. The different concentrations of the drug are indicated. b. Maximal growth rates obtained in the negative assay. c. Positive assay in which SARS-CoV-2 E protein is expressed at a low level (induced with 20 μM [β-D-1-thiogalactopyranoside]) in K^+-uptake deficient bacteria [31]. In this instance, inhibitory drugs reduce bacterial growth. d. Maximal growth rates obtained in the positive assay. e. Fluorescence-based conductivity assay. The fluorescence of bacteria that harbor a pH-sensitive GFP [32] and express the SARS-CoV-2 E protein was examined as a function of different chemical concnetration as noted. The experiment was performed as previously described [33], whereby at time 0, a concentrated solution of citric acid was injected into the media. In all panels LB indicates bacteria that do not express the channel as a positive control, while 100 μM IPTG indicates no drug added as a negative control.

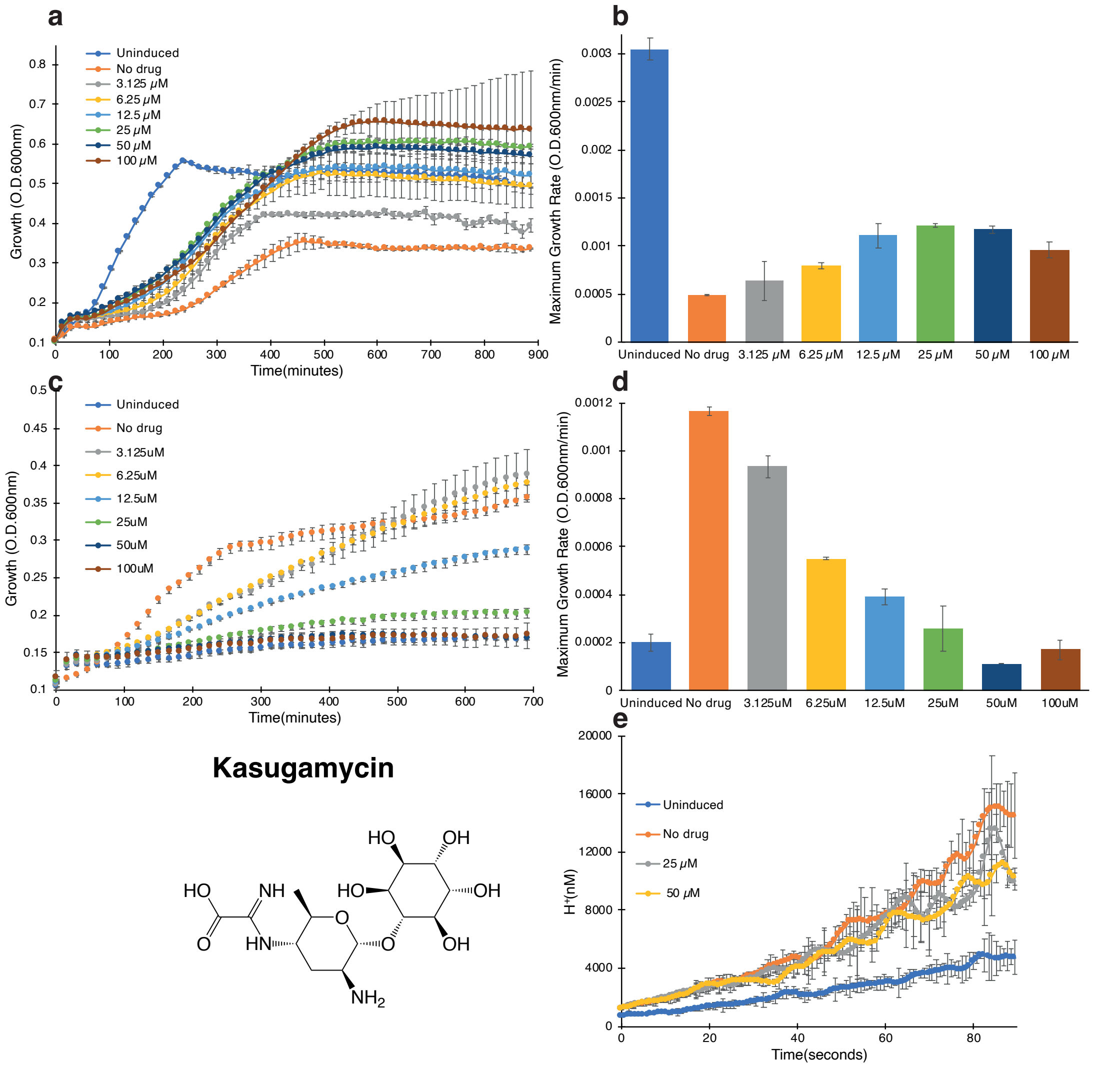

Figure S7. Raw screening data for Kasugamycin a. Negative assay in which SARS-CoV-2 E protein is expressed at an elevated level (induced with 100 μM [β-D-1-thio-galactopyranoside]) and is therefore deleterious to bacteria. The different concentrations of the drug are indicated. b. Maximal growth rates obtained in the negative assay. c. Positive assay in which SARS-CoV-2 E protein is expressed at a low level (induced with 20 μM [β-D-1-thiogalactopyranoside]) in K^+-uptake deficient bacteria [31]. In this instance, inhibitory drugs reduce bacterial growth. d. Maximal growth rates obtained in the positive assay. e. Fluorescence-based conductivity assay. The fluorescence of bacteria that harbor a pH-sensitive GFP [32] and express the SARS-CoV-2 E protein was examined as a function of different chemical concnetration as noted. The experiment was performed as previously described [33], whereby at time 0, a concentrated solution of citric acid was injected into the media. In all panels LB indicates bacteria that do not express the channel as a positive control, while 100 μM IPTG indicates no drug added as a negative control.

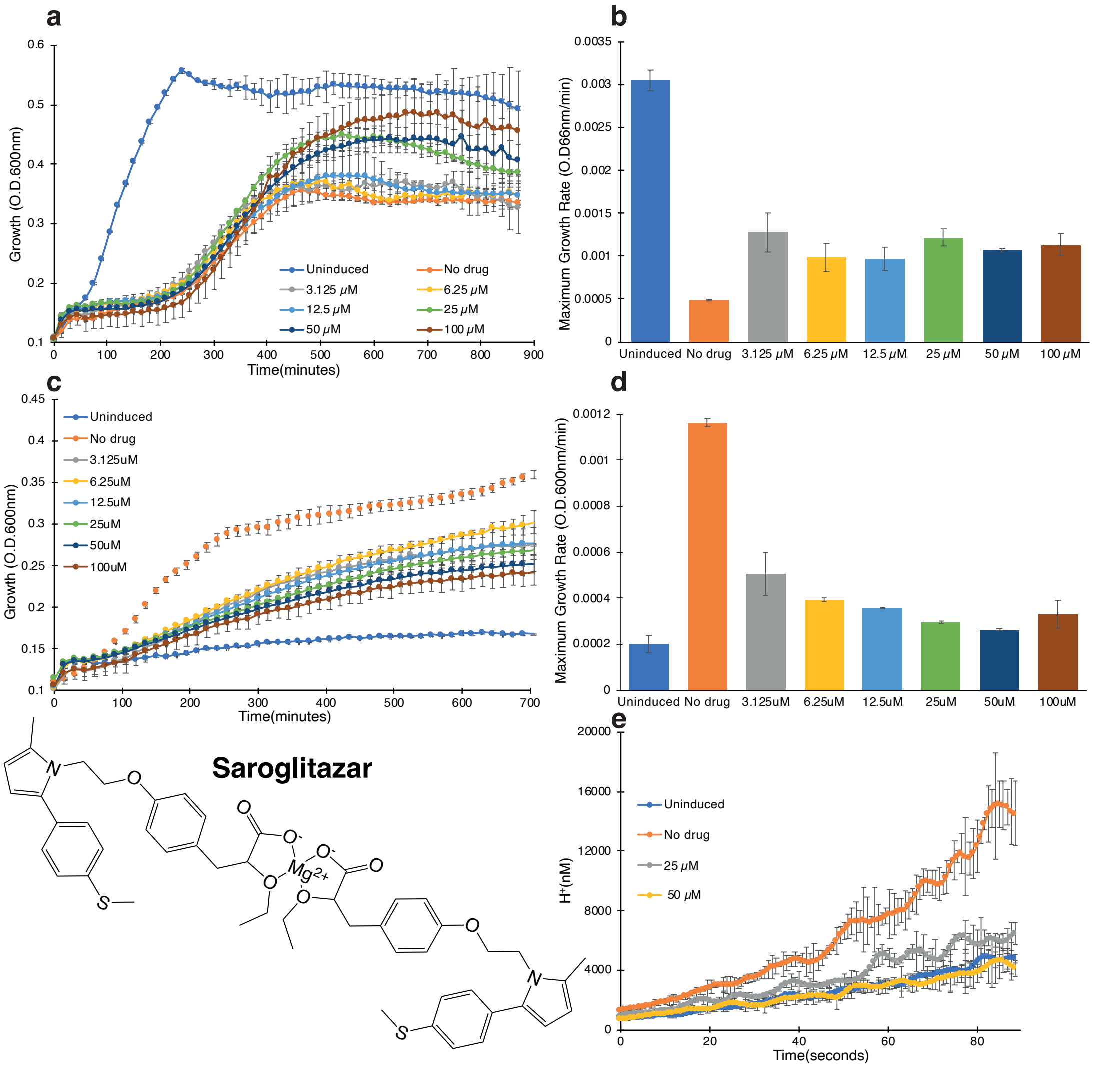

Figure S8. Raw screening data for Saroglitazar. a. Negative assay in which SARS-CoV-2 E protein is expressed at an elevated level (induced with 100 μM [β-D-1-thiogalactopyranoside]) and is therefore deleterious to bacteria. The different concentrations of the drug are indicated. b. Maximal growth rates obtained in the negative assay. c. Positive assay in which SARS-CoV-2 E protein is expressed at a low level (induced with 20 μM [β-D-1-thiogalactopyranoside]) in K^+-uptake deficient bacteria [31]. In this instance, inhibitory drugs reduce bacterial growth. d. Maximal growth rates obtained in the positive assay. e. Fluorescence-based conductivity assay. The fluorescence of bacteria that harbor a pH-sensitive GFP [32] and express the SARS-CoV-2 E protein was examined as a function of different chemical concnetration as noted. The experiment was performed as previously described [33], whereby at time 0, a concentrated solution of citric acid was injected into the media. In all panels LB indicates bacteria that do not express the channel as a positive control, while 100 μM IPTG indicates no drug added as a negative control.
